# Deregulations in the Cyclin-Dependent Kinase-9-Related Pathway in Cancer: Implications for Drug Discovery and Development

**DOI:** 10.1155/2013/305371

**Published:** 2013-06-06

**Authors:** Gaetano Romano

**Affiliations:** College of Science and Technology, Department of Biology, Temple University, Bio Life Science Building, Suite 456, 1900 North 12th Street, Philadelphia, PA 19122, USA

## Abstract

The CDK9-related pathway is an important regulator of mammalian cell biology and is also involved in the replication cycle of several viruses, including the human immunodeficiency virus type 1. CDK9 is present in two isoforms termed CDK9-42 and CDK9-55 that bind noncovalently type T cyclins and cyclin K. This association forms a heterodimer, where CDK9 carries the enzymatic site and the cyclin partner functions as a regulatory subunit. This heterodimer is the main component of the positive transcription elongation factor b, which stabilizes RNA elongation via phosphorylation of the RNA pol II carboxyl terminal domain. Abnormal activities in the CDK9-related pathway were observed in human malignancies and cardiac hypertrophies. Thus, the elucidation of the CDK9 pathway deregulations may provide useful insights into the pathogenesis and progression of human malignancies, cardiac hypertrophy, AIDS and other viral-related maladies. These studies may lead to the improvement of kinase inhibitors for the treatment of the previously mentioned pathological conditions. This review describes the CDK9-related pathway deregulations in malignancies and the development of kinase inhibitors in cancer therapy, which can be classified into three categories: antagonists that block the ATP binding site of the catalytic domain, allosteric inhibitors, and small molecules that disrupt protein-protein interactions.

## 1. Introduction

**Figure 1:**
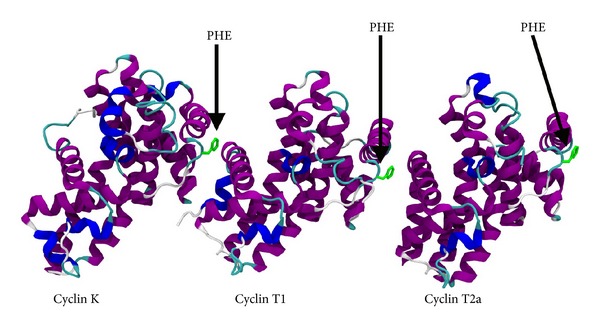
This figure displays the binding partners for Cdk9: cyclin K, cyclin T1, and cyclin T2a. Cyclin T2b is very similar to cyclin T2a and is not shown. Abbreviation: PHE: phenylalanine.

**Figure 2:**
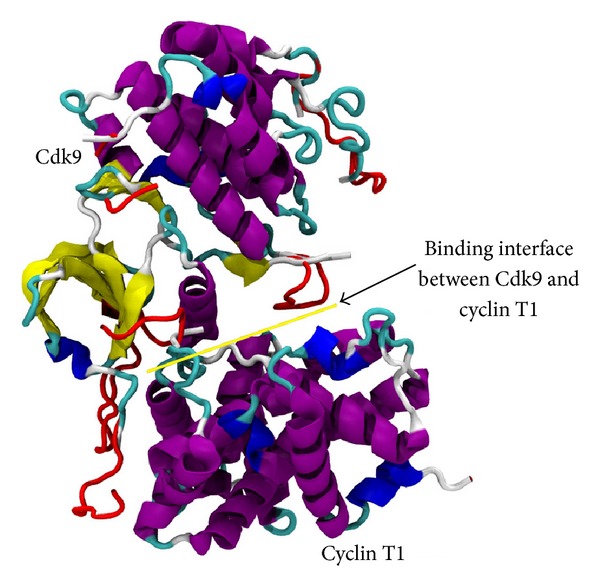
The Cdk9/cyclin T1 complex. The arrow depicts a yellow line, which shows the binding interface between Cdk9 and cyclin T1.

**Figure 3:**
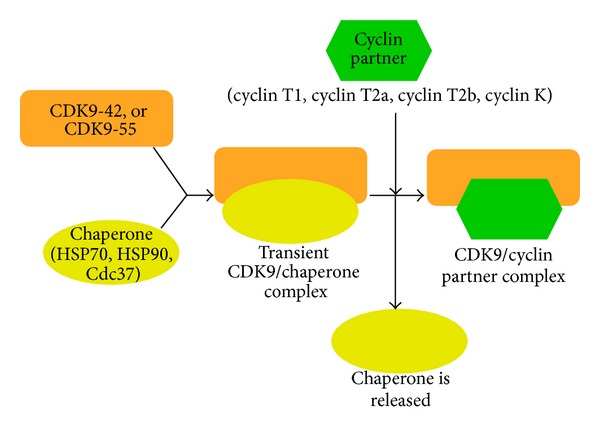
The components of the CDK9-related pathway.

Cyclin-dependent kinase 9 (CDK9) is a member of the cdc2-like serine/threonine kinase family and was identified in the early 1990s [1–3]. At that time, CDK9 was initially named PITALRE [1–3]. Overall, the Cdk9-related pathway comprises two isoforms termed CDK9-42 and CDK9-55 and four cyclin partners, such as cyclin T1, cyclin T2a, cyclin T2b, and cyclin K (Figure 1) (Table 1) [4–10]. The denominations 42 and 55 are related to the apparent molecular weight of the two CDK9 isoforms that were observed in Western blot analysis [4–10]. The noncovalent association between CDK9 and one of its cyclin partners gives rise to a heterodimer, in which CDK9 carries the enzymatic site and the cyclin functions as a regulatory subunit (Figure 2) [1–10]. The establishment of the heterodimer is essential to confer biological activity to the CDK9/cyclin partner complex [1–10]. The latter is quite stable [11]. This is in contrast to the monomeric CDK9, which is susceptible to a rather fast degradation [12]. For this reason, monomeric CDK9 molecules must form a transient complex with chaperone proteins HSP70, HSP90, and Cdc37, prior to the final association with the corresponding cyclin partners (Figure 3) [12]. 

CDKs are essential factors in mammalian cell biology (Table 2). Many CDKs are directly involved in the regulation of cell cycle, such as CDK1, CDK2, CDK3, CDK4, CDK6, and CDK11 [13, 14]. 

In contrast to all the other CDKs, CDK5 relies on the noncyclin partners p35 and p38 [15–17] and performs an important role in the orchestration of cellular senescence [13, 18–20], survival of neurons [15], neuronal death [15, 17, 21, 22], formation of dendrite synapses and extensions [15, 16], migration of neurons into the granule cell layer [15, 16], and inhibition of cell cycle reentry in postmitotic neurons [15]. 

CDK7, CDK8, and CDK9 are involved with the control of RNA-polymerase-II- (pol II-) mediated transcription [3, 10, 13, 23–28]. In addition, CDK7 acts as a CDK-activating kinase (CAK) [13]. A recent study conducted on a human glioblastoma cell line showed that protein kinase C- (PKC-) *ι* phosphorylates CDK7 at Thr170 and CDK2 at Thr160 [14]. This finding indicates that human glioblastoma cells may proliferate via a novel PI (3)-kinase-/PKC-*ι*/Cdk7/cdk2-mediated pathway [14]. Interestingly, CDK7 has the ability to phosphorylate the human estrogen receptor-*α* (ER*α*) at Ser294, which is one of the hallmarks of ER*α* activation in human breast cancer [29]. The aspects related to the CDK-mediated control of RNA pol II transcription will be discussed in greater detail in the following section, together with various factors that interact with the CDK9-related pathway.

 CDK10 controls its own transcriptional activity through the association with the C-ets-2 transcription factor and takes part in the regulation of the G_2_-M cell cycle phase [30–33]. Recent findings seem to indicate that CDK10 might act as a putative tumor suppressor gene [34, 35] and that a reduced CDK10 expression is quite likely linked with the development and progression of hepatocellular carcinoma [35].

 CDK11 regulates cell cycle progression, RNA-pol-II-mediated transcription, splicing of premessenger RNA, and centriole duplication [13, 36–41]. 

 In addition to CDK9, cyclin K associates with CDK12 and CDK13 [42–44]. These heterodimers are both implicated in the control of RNA-pol-II-mediated transcription [42–44]. CDK12 and CDK13 may also associate with L-type cyclins and take part in the regulation of alternative RNA splicing [45, 46]. 

 CDK14 is also known either as PFTK1 or PFTAIRE1 and regulates cell cycle progression and cell proliferation [47–50]. CDK14 can associate with D-type cyclins [47–50] and cyclin Y [51, 52]. Recent reports have demonstrated that CDK14 increases cell motility in human hepatocellular carcinoma cells [53, 54]. Moreover, higher levels of CDK14 expression are correlated with enhanced chemoresistance in human primary oesophageal squamous cell carcinoma cells [55]. 

 The CDK9-related pathway regulates a wide range of functions in mammalian cell biology [1–12, 24] and the replication program of numerous viral agents, such as the human immunodeficiency virus type 1 (HIV-1) and HIV-2 [25, 56], Epstein-Barr virus (EBV) [57], human T-lymphotropic virus type 1 (HTLV-1) [58, 59], human cytomegalovirus (hCMV) [60, 61], herpes simplex virus 1 (HSV-1) [62, 63], human adenovirus [64], and influenza A virus [65] (Table 2). Moreover, dysfunctions in the CDK9-related pathway are related with several forms of human tumors [9, 13, 26–28, 66–68] and cardiac hypertrophy [69–75]. So far, many CDK inhibitors have been applied in clinical trials for the treatment of various malignancies (Table 3) [13, 26–28]. Studies are currently ongoing to evaluate the possibility of using CDK inhibitors in clinical trials for the treatment of AIDS, malaria, cardiac hypertrophy, type 2 diabetes, inflammatory kidney disease, and neurological disorders [13]. 

 This paper focuses on the relevance of the deregulations in CDK9-related pathway in cancer and on the standpoint of the discovery and development of CDK inhibitors for cancer therapy.

## 2. Biological Functions of the CDK9-Related Pathway

 The CDK9-related pathway is one of the primary factors in the regulation of mammalian cell biology [9, 13, 25, 27, 28]. Studies in the human and murine systems showed that CDK9 is present in all kinds of tissues and is highly expressed in terminally differentiated cells [2, 3, 9]. As anticipated, there are two CDK9 isoforms in mammalian cells: CDK9-42 and CDK9-55 [4–6]. The CDK9-55 isoform has 117 additional amino acid residues in front of the amino terminus of CDK9-42 [4]. The genetic organization of the human CDK9 promoter comprises two transcription starts [4, 5]. The human CDK9 promoter that encodes for CDK9-42 mRNA does not have a functional TATA box and contains a GC-rich sequence, and the region −352 to −1 includes the required transcriptional elements to sustain full promoter activity. These factors result in constitutive high levels of human CDK9-42 promoter activity, which is similar to a housekeeping gene promoter [7]. In contrast, the human CDK9-55 promoter contains a TATA box, which is situated approximately 500 bp upstream of the human CDK9-42 transcription start [4, 7]. 

 The CDK9-42 isoform is present both in the cell cytoplasm and nucleus [12], whereas CDK9-55 is predominantly localized in the cell nucleus [4]. Differential patterns of tissue distribution in the human and mouse model were observed between the two CDK9 isoforms [4, 5]. For instance, CDK9-42 expression has a bias for testis and spleen, whereas CDK9-55 is predominantly found in liver tissues, brain, and lung [4, 5]. Similar findings in differential patterns of expression were observed in human and mouse cell culture systems. On one hand, CDK9-42 is primarily expressed in human cervical carcinoma HeLa cell line [76], human primary undifferentiated monocytes [8], and mouse NIH/3T3 fibroblasts [4]. On the other hand, CDK9-55 expression becomes predominant after induced differentiation of human primary monocytes along the macrophage lineage [8]. A considerable decline in CDK9-55 expression was reported following the activation of human lymphocytes, in which CDK9-42 becomes the predominant form [8]. Another study showed that CDK9-55 is primarily expressed in rat hepatocytes, when these cells are inside the liver, but CDK9-42 surmounts CDK9-55 expression once the rat hepatocytes are established in primary cell cultures [8]. Thus, the two CDK9 isoforms are differentially expressed depending on the cell signaling system and/or cell type. Lastly, several studies showed that CDK9-55 is associated with the regulation of the cell differentiation program of various tissues, such as the hematopoietic compartment [4, 5, 8], the muscle [68, 77], and adipogenesis [78]. 

**Figure 4:**
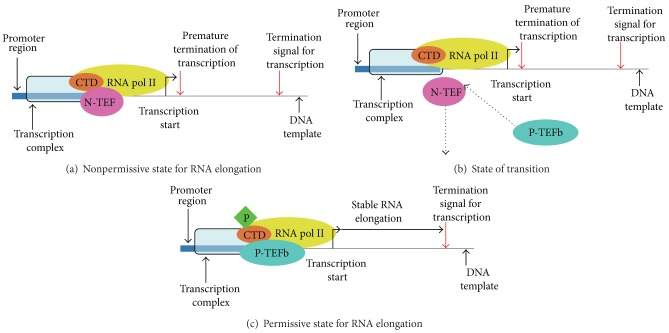
This figure describes the role of the P-TEFb complex in stabilizing RNA elongation. Panel (a) illustrates a nonpermissive state for RNA elongation. In this configuration, RNA pol II is associated with N-TEF, and the RNA pol II CTD is not phosphorylated. In this case, RNA transcription can start but stops prematurely. Panel (b) shows a state of transition, in which P-TEFb is about to substitute N-TEF in the transcription complex. P-TEFb contains the complex Cdk9/cyclin partner. Panel (c) exhibits a permissive state for RNA elongation. The presence of P-TEFb in the transcription complex phosphorylates the RNA pol II CTD, which, in turn, allows for the stabilization of the RNA transcript elongation. Abbreviations: RNA pol II: RNA polymerase II; N-TEF: negative transcription factor; CTD: carboxyl terminal domain of RNA pol II; P-TEFb: positive transcription factor b.

 Both CDK9 isoforms can combine either with type T cyclins or cyclin K to generate a heterodimer (Tables 1 and 2), which is the principal constituent of the positive transcription elongation factor b (P-TEFb) (Figure 4) [5, 8, 25, 79–82]. The P-TEFb complex phosphorylates the carboxyl-terminus domain (CTD) of the RNA pol II, which, in turn, stabilizes the elongation of the RNA transcript [25, 80–82]. The kinase activity of P-TEFb is specifically inhibited by 5,6-dichloro-1-*β*-D-ribofuranosylbenzimidazole (DRB) [5, 8, 25]. The nonphosphorylated RNA pol II can start RNA transcription in the initial stages of the transcription complex assembly but is not able to support the full elongation of the nascent RNA transcript (Figure 4) [25, 80, 83]. This is due to the association between RNA pol II and the negative transcription elongation factor (N-TEF) (Figure 4) [80, 83]. The presence of the P-TEFb accomplishes a dual function: the ejection of N-TEF from the transcription complex and the subsequent phosphorylation of the RNA pol II CTD, which becomes effective in maintaining the elongation of the nascent RNA transcript (Figure 4) [25, 80, 83]. The human RNA pol II CTD contains 52 repeats of the heptapeptide Y_1_S_2_P_3_T_4_S_5_P_6_S_7_ [84–86]. The nonphosphorylated RNA pol II is firmly linked with the various factors of the preinitiation complex, which include the TATA-binding protein (TBP) and the mediator complex [84–86]. A study conducted in the *in vitro* system showed that the CDK7-mediated phosphorylation of Ser5 of the heptapeptide reduces the binding affinity between the RNA pol II CTD and the other components of the preinitiation complex [84]. Then, the capping enzyme binds to the phosphorylated Ser5 CTD, and the RNA capping structure is associated with the nascent RNA transcript, which is approximately 25–30 nucleotides long [84]. However, Ser5 phosphorylation of RNA pol II CTD is not *per se* sufficient to stabilize the elongation of the RNA transcript, which also needs the CDK9-mediated phosphorylation of Ser2 of the CTD heptapeptide [84]. Interestingly, an *in vivo* study showed that CDK7 and CDK9 were able to substitute each other in the phosphorylation of Ser5 and Ser2 of the RNA pol II CTD [87], which is in contrast with the observation of the *in vitro* model [84, 87]. This study was conducted on human glioblastoma and human prostate cancer cell lines and utilized siRNA molecules for the silencing of CDK9- and/or CDK7-related pathways [87]. The biological efficacy of the silencing of the two pathways was monitored by Western blot assay with antibodies that were specific either for phosphorylated Ser2 or phosphorylated Ser5 of the RNA pol II CTD [87]. The best inhibition of Ser2 and Ser5 phosphorylation required the simultaneous silencing of CDK9 and CDK7 [87]. Indeed, this finding may have significant implications both for the design of kinase inhibitors in cancer therapy and the development of a platform for microarray analysis of the two signaling systems in malignant cells [87].

 The phosphorylation of the RNA pol II CTD is crucial for the CDK9-related pathway regulation of gene expression in mammalian cell biology [13, 25, 27]. Interestingly, the CDK9-related pathway also interacts with several other cellular and viral factors (Table 2) [3, 10, 12, 13, 24–28, 56–67, 88–107]. These interactions may consist of phosphorylation and/or physical association between the CDK9-related pathway and various cellular or viral components. Some of these factors comprise MyoD, p53, pRb, cMyc, hSPT5, TRAF2, STAT3, SkiP, NK-*κ*B, BRD4, SMAD, UBE2A, NELF-E, HEXIM1, HEXIM2, 7SK snRNA, gp130, HSP70, HSP90, Cdc37, E12/E47, hCMV UL69, EBV EBNA-2, HSV-1 ICP22 and ICP27, HIV-1, and HIV-2 Tat protein (Table 2). As anticipated, the chaperone proteins HSP70, HSP90, and Cdc37 bind transiently and, therefore, stabilize the monomeric CDK9 preceding the association with one of its cyclin partners [11, 12]. Interestingly, monomeric CDK9 has the ability to bind to the cytoplasmic region of the receptor for the interleukin-6 (IL-6) family of cytokines, which is termed gp130 [12, 98]. Such a phenomenon indicates a probable involvement of CDK9 as an intermediary in the regulation of the IL-6-related signal transduction pathway [12, 98]. 

 The CDK9-mediated posttranslational modification of cellular factors constitutes an additional prospect for the genetic regulation of mammalian cell biology [13, 25, 27]. The same applies for the phosphorylation and/or association between the CDK9-related pathway and viral factors, which, once they are modified, take part in the gene regulation of the viral agent within the host cell [13, 25, 27]. 

 In addition, the CDK9-related pathway arbitrates the regulation of the cotranscriptional mRNA processing, chromatin modification, activation of quiescent B and/or T lymphocytes, cell differentiation, and cell survival (Table 2) [24, 66, 67, 78, 79, 85, 99–106]. The CDK9-related pathway is a main player in the regulation of the differentiation program in the hematopoietic compartment [4, 5, 8], muscle tissues [77, 107], central nervous system [67], and adipogenesis [78]. There is a great deal of interest in the involvement of the CDK9-related pathway and cell survival and/or protection from apoptosis. Previous studies demonstrated that the overexpression of a dominant negative CDK9 (CDK9-dn) increased susceptibility to apoptosis in human monocytes [12], human U-937 promonocytic cells, and human Jurkat T cell line [100]. 

 Indeed, the CDK9-related signaling system is an important pathway in mammalian cell biology and covers a wide range of functions, which comprise cell survival and protection from apoptotic injuries. The latter might have important implication in the context of a deregulated CDK9 pathway in the establishment and maintenance of a transformed cell phenotype. 

## 3. The Deregulated CDK9 Pathway in Cancer

 Tumorigenesis is a multistep process, which requires a combination of genetic and epigenetic mutations, silencing of tumor suppressor genes, activation and/or overexpression of oncogenes, and environmental factors [108–113]. Several studies reported a connection between malignant cell transformation and abnormal antiapoptotic signaling systems, such as epidermal growth factor receptor (EGF-R), insulin-like growth factor-I receptor (IGF-1R), AKT-related pathway, autocrine/paracrine secreted Frizzled-related protein 2, and survivin [114–121]. In this respect, additional studies showed that a deregulated CDK9 signaling system might also have important implications in the development and/or maintenance of a malignant cell phenotype [9, 66, 67, 78, 100, 122]. Aberrant patterns of cellular protein phosphorylation are indicative of hyperactive protein kinase pathways and are often observed both in tumors and many other maladies, such as neurological diseases, diabetes, inflammations, and infections [13, 26–28, 123, 124]. In particular, a deregulated CDK9 pathway increases the expression of myeloid leukemia cell differentiation protein (Mcl-1), as shown by studies on biopsies obtained from patients with either advanced chronic lymphocytic leukemia or multiple myeloma [122, 125–127]. Furthermore, deregulations in the CDK9-related pathway were reported in a number of human malignancies, such as lymphomas [66, 128], neuroblastoma [67], primary neuroectodermal tumor [67], rhabdomyosarcoma [68], and prostate cancer [129]. 

 Increased expression levels of CDK9 and cyclin T1 were observed in B and T cell precursor-derived lymphomas, anaplastic large T cell lymphoma, and follicular lymphomas, while Hodgkin and Reed-Sternberg cells of classical Hodgkin's lymphoma were characterized by a strong nuclear staining for both proteins [66]. In addition, abnormal mRNA levels of CDK9 and cyclin T1 were found in Burkitt's lymphoma, diffuse large B cell lymphoma with germinal center phenotype, classical Hodgkin's lymphoma-derived cell lines, and follicular lymphoma [66].

 These findings, taken together, indicate that CDK9, along with other CDKs, might be a suitable therapeutic target for cancer therapy. 

## 4. The Standpoint of Protein Kinase Development for Cancer Therapy

 The pharmacological inhibition of CDKs for the treatment of malignancies has indeed attracted a great deal of interest over the last years [13, 26–28]. The majority of protein kinase inhibitors are nucleoside analogs and aim at neutralizing the adenosine triphosphate (ATP) binding site situated in the CDK enzymatic moiety [130–133]. Many CDK inhibitors have been utilized in phase I and phase II clinical trials for the treatment of an ample variety of cancers (Table 3). In this respect, seliciclib and alvocidib belong to the first generation of CDK inhibitors and have been utilized in phase I and phase II clinical trials for the treatment of many types of tumors [13, 26–28]. However, these clinical trials reported a modest clinical efficacy in oncological patients treated either with seliciclib or alvocidib [13, 26–28]. 

 The so-called first generation of CDK inhibitors is characterized by a wide-ranging anti-CDK activity and target at the same time CDK1, CDK2, CDK4, CDK6, CDK7, and CDK9 [13, 26–28]. In addition to seliciclib and alvocidib, the first generation of CDK inhibitors includes dinaciclib (or SCH727965), SNS-032 (or BMS-387032), AG-024322, and R-547 (or R_0_-4584820) (Table 3) [13, 26–28, 134].

 The activity of the second generation of CDK inhibitors tends to aim at a more circumscribed group of CDKs, which usually comprises CDK4, CDK6, and/or CDK2 (Table 3) [13, 26–28]. The second generation of CDK inhibitors includes PD-0332991, P276-00, AT-7519, and RGB-286638 (Table 3) [13, 26–28, 135–144]. 

 Lastly, the third generation of CDK inhibitors is directed against a number of CDKs, along with other types of protein kinases [13, 26–28, 134]. This strategy attempts to optimize the antitumor efficacy of the third generation of CDK inhibitors, which includes ZK 304709, GPC-286199 (or RGB-286199) and JNJ-7706621 (Table 3) [13, 26–28, 145–149]. For instance, the compound ZK 304709 was utilized in phase I clinical trials for the treatment of patients with refractory and/or relapsed solid tumors and inhibits CDK1, CDK2, CDK4, CDK7, CDK9, vascular endothelial growth factor receptors VEGFR-1, VEGFR-2, and VEGFR-3, platelet-derived growth factor receptor *β* (PDGFR-*β*), and Flt3 (Table 3) [145, 146]. GPC-286199 inhibits CDK1, CDK2, CDK3, CDK5, CDK7, CDK9, and CRK-related kinases [13, 26–28, 150, 151], whereas JNJ-7706621 targets CDK1, CDK2, CDK3, and Aurora-A/B kinases (Table 3) [13, 26–28, 147–149]. Both GPC-286199 and JNJ-7706621 are still at a preclinical stage testing. 

## 5. Future Directions for Drug Discovery and Development in Cancer Therapy

 As already mentioned, most of protein kinase inhibitors are nucleoside analogs and were designed to block the ATP binding site of the catalytic domain of various CDKs, such as alvocidib (or flavopiridol) and seliciclib [13, 26–28]. However, this therapeutic approach is associated with toxicity [13]. In addition, alvocidib and seliciclib exhibited a modest pharmacological activity in clinical trials for the treatment of patients with different kinds of tumors [13]. Several reasons may account for the toxicity of the two compounds [13]. One of these reasons might be related to the targeting of the ATP binding site of the CDK enzymatic domain, which has a conserved structure among protein kinases [152]. Thus, nucleoside analog CDK inhibitors become responsible for off-target effects, which may impair to some extent the normal functions of other types of protein kinases and, as a result, cause a considerable harm in normal cells and/or tissues [152]. In order to circumvent this issue, the field of drug design is developing the so-called allosteric inhibitors, which either modify the protein kinase conformation to inhibit the function of the CDK ATP binding site [153] or compete directly with the binding of the regulatory subunit of the kinase to the protein substrate [153–157]. Some examples of allosteric kinase inhibitors that impair ATP binding to the catalytic site comprise Gleevec (imatinib mesylate), BIRB796 (Doramapimod), BAY43-9006 (Sorafenib or Nexavar), and AAL-993 [153]. Gleevec was designed to inhibit many types of tyrosine kinases, such as Bcr-Abl, stem cell factor receptor (c-Kit), and platelet-derived growth factor receptor (PDGF-R) [153]. BIRB796 targets serine/threonine kinase p38 MAPK [153]. BAY43-9006 is a Raf inhibitor, whereas AAL-993 is an antiangiogenic factor that suppresses the kinase activity of VEGF-1R, VEGF-R2, and VEGF-R3 [153]. Other so-called ATP noncompetitive allosteric inhibitors that interfere with the interaction between the regulatory subunit of the kinase and the protein substrate include PD09859, CMPD1, Coumarin derivatives G8935 and G0328, API-2, amino-functionalized quinoxaline 5, Pyrazinone derivative 14f, Akt-I-1, Thiadiazolines, Chloromethyl thienylketone 17, and BMS-345541 [153]. Specifically, PD09859 is an MEK inhibitor, whereas CMPD1 and Coumarin derivatives G8935 and G0328 target the extracellular signal regulated kinase (ERK) mitogen-activated protein kinases (MAPKs) [153]. The AKT pathway is inhibited by API-2, amino-functionalized quinoxaline 5, Pyrazinone derivative 14f, and Akt-I-1. Thiadiazolines and Chloromethyl thienylketone 17 impair the functions of Glycogen synthase kinase 3-*β* [153]. Lastly, BMS-345541 competes with the binding between the protein substrate and threonine/serine I*κ*B kinases [153]. 

 Interestingly, other classes of protein kinase inhibitors are currently under development for the targeting of wide interfaces between two proteins [158–165]. On one hand, this approach holds great therapeutic potential, but, on the other hand, the formulation of molecules that have the ability to disrupt the interaction between two proteins poses a daunting challenge. In fact, the contact surfaces between two interacting proteins may be in the range of 1,500 to 3,000 A^2^ [166, 167], whereas the interactions between a protein and a small molecule typically involve contact surfaces that may vary from 300 to 1,000 A^2^ [168, 169]. Furthermore, contact interfaces between two interacting proteins are usually flat and do not have pockets and/or grooves, which may be quite normally found on surfaces of proteins that bind to small molecules [170]. However, several mutational studies showed that a small number of amino acid residues involved in holding together protein-protein interface account for most of the free energy for the binding [171–175]. These so-called hotspots are usually found at the center of the contact interface and take less than half of the total contact interface implicated in protein-protein interactions. For example, the binding interface between CDK9 and cyclin T1 is flat and hydrophobic (Figure 2) [176]. The hydrophobic residues that constitute the hydrophobic pocket in the binding region of CDK9 and cyclin T1 comprise Leucine 64 (CDK9), Phenylalanine 59 (CDK9), Isoleucine 67 (CDK9), Isoleucine 84 (CDK9), Isoleucine 99 (CDK9), and Phenylalanine 146 (cyclin T1). Phenylalanine 146 residue of cyclin T1 is also present among the other cyclin partners of CDK9, such as cyclin T2a, cyclin T2b, and cyclin K (Figure 1) [176]. Remarkably, studies conducted on Iron chelators ICL670 and ICL311 reported kinase activity inhibition following the separation of CDK9 from cyclin T1, which, in turn, resulted either in suppression of HIV-1 replication [164, 165] or repression of tumor growth *in vitro* and in animal models [177]. The exact mechanism of Iron chelator-mediated dissociation of the CDK9 and cyclin T1 complex is still under investigation. 

 As indicated by the studies on the binding interface between CDK9 and cyclin T1, almost identical hotspot regions might be in common among other protein-protein interacting surfaces, such as CDK7 and cyclin H, for example, [178, 179]. Other reports described the presence of promiscuous contact surfaces in a number of protein-protein interactions [180]. Therefore, the design of small molecules for the inhibition of protein-protein interactions is always susceptible to undesired off-target effects. Nevertheless, encouraging achievements were reported in designing small molecules that exhibited specific targeting for protein-protein interactions [181–184]. A number of inhibitors for protein-protein interfaces were developed for interleukin-2 (IL-2), Bcl-X_*L*_, human protein double minute 2 (HDM2), human papilloma virus (HPV) transcription factor E2, membrane-anchored bacterial ZipA protein, and binding of tumor necrosis factor (TNF) to its receptors (TNFR1 and TNFR2) [158]. Other studies are aiming at producing new classes of macromolecules termed “bis-peptides”, which have molecular weights ranging from 750 to 2000 Da [185, 186]. Such bis-peptides have the ability to create large, extended preorganized surfaces, which may provide outstanding drug characteristics for the disruption of protein-protein interfaces [185, 186]. This approach allowed for the synthesis of a functionalized bis-peptide that inhibited the ubiquitination of wild-type p53 in human liver cancer cell lines [187]. The bis-peptide reproduced the activation domain of the tumor suppressor gene p53 and was utilized to neutralize HDM2 in human liver cancer cell lines [187]. HDM2 is a human E3 ubiquitin ligase and a suppressor of p53 expression through ubiquitination. Therefore, the bis-peptide-mediated disruption of HDM2/p53 binding caused the inhibition of wild-type p53 ubiquitination [187, 188]. The accumulation of wild-type p53 may subsequently trigger apoptosis in malignant cells [187, 188]. 

## 6. Conclusion

The CDK9-related pathway has emerged as a target of extreme for cancer therapy. Deregulations in this pathway were observed in a variety of human tumors, which may induce increased expression and/or hyperactivity of cellular oncogenic factors. In fact, the use of kinase inhibitor in clinical trials for the treatment of patients with chronic lymphocytic leukemia showed that the inhibition of CDK9- and CDK7-related pathways was responsible for the decrease of the antiapoptotic factor Mcl-1 [125–127]. On these grounds, there is a keen interest in producing new compounds with enhanced specificity for CDK9- and/or CDK7-related pathways. 

The field of drug design is currently striving to improve the therapeutic index of kinase inhibitors for cancer therapy, in order to minimize the toxicity associated with kinase inhibitors that block the ATP binding site of the catalytic domain of the enzyme. In this respect, drug designers are pursuing two main strategies: the engineering of allosteric kinase inhibitors and of inhibitors that disrupt protein-protein interactions. 

## Figures and Tables

**Table 1:** Schematic representation of the Cdk9-related pathway.

Cdk9 isoforms	Cdk9-42; Cdk9-55
Cyclin partners	Cyclin T1; cyclin T2a; cyclin T2b; cyclin K
Chaperone proteins	HSP70; HSP90; Cdc37

**Table 2:** Properties of cyclin-dependent kinases and binding partners in mammalian cell biology.

Cyclin-dependent kinases (Cdks)	Most important binding partners for Cdks (secondary binding partners are indicated between parenthesis)	List of major factors that interact with the heterodimer Cdk/cyclin partner	Functions of the heterodimer Cdk/cyclin partner in mammalian cell biology	Bibliographic references
Cdk1	Cyclins A1; A2; B1; B2; (E; B3)	Cell cycle machinery; Cdc28-dependent kinase subunit (Cks)	G2-M (cell cycle)	[13]

Cdk2	Cyclins A1; A2; E1; E2 (D1; D2; B1; B3)	Cell cycle machinery; protein kinase C-(PKC)-*ι*	G1-S (cell cycle)	[13, 14]

Cdk3	Cyclins E1; E2; A1; A2; C	Cell cycle machinery; E2F/dimerization partner (DP)	G0-G1-S (cell cycle)	[13]

Cdk4	Cyclins D1; D2; D3	Cell cycle machinery; MyoD	G1-S (cell cycle)	[13]

Cdk5	p35; p39 (cyclins D; E; G)	—	Senescence; inhibition of cell cycle reentry in postmitotic neurons; neuronal migration; formation of dendrite extensions and synapses; neuronal survival; neuronal death	[13–22]

Cdk6	Cyclins D1; D2; D3	Cell cycle machinery	G1-S (cell cycle)	[13]

Cdk7	Cyclin H and MAT	RNA pol II; Protein kinase C- (PKC-) *ι*; estrogen receptor-*α*	Transcription; Cdk-activating kinase (CAK)	[13, 14, 26–29]

Cdk8	Cyclin C (K?)	RNA pol II; Smad	Transcription	[23]

Cdk9	Cyclins T1; T2a; T2b; K	RNA pol II; MyoD; p53; retinoblastomas gene (pRb); hSPT5, c-Myc; SkiP; Smad; signal transducer and activator of transcription 3 (STAT3); TRAF2; Brd4; NF-*κ*B; Suppressor of Ty Homolog-5 (SUPT5H); Negative Elongation Factor-E (NELF-E); human Rad6 homolog UBE2A; E12/E47 (members of the basic helix-loop-helix family); Hexamethylene bisacetamide-inducible protein 1 (Hexim1); Hexim2; 7SK snRNA; p300/GATA4, HIV-1 and HIV-2 Tat protein; HTLV-1 tax protein; EBV EBNA2; hCMV pUL69; hCMV IE2; HSV-1 ICP22 and ICP27; adenovirus large E1A (L-E1A) protein; influenza virus RNA-dependent RNA polymerase (vRNP); gp130*; HSP70*; HSP90*; Cdc37* (*these factors form a transient association with the monomeric Cdk9)	Transcription; cotranscriptional mRNA processing; regulation of chromatin modification; cell differentiation (B and T lymphocytes, muscle cells); adipogenesis; protection from apoptosis	[3, 10, 12, 13, 24–28, 56–67, 78, 79, 85, 88–107]

Cdk10	Unidentified	Ets2	Transcription; G2M (cell cycle)	[30–35]

Cdk11	Cyclins L1; L2; (D)	Cell cycle machinery; RNA pol II; RanBPM; RNPS1; CK2; 14-3-3; 9G8; elF3; NOT2; HBO1	Transcription; pre-mRNA splicing; M (cell cycle)	[13, 36–41]

Cdk12	Cyclins L1; L2; K	RNA pol II;	Transcription; regulation of RNA splicing; genome stability	[42–46]

Cdk13	Cyclins L1; L2; K	RNA pol II;	Transcription; regulation of RNA splicing	[42–46]

Cdk14	Cyclins D1; D2; D3; Y	—	Cell cycle progression; cell proliferation; cell motility; chemoresistance in human primary oesophageal squamous cell carcinoma cells	[47–55]

**Table 3:** CDK inhibitors utilized in clinical trials for the treatment of various types of malignancies (http://www.clinicaltrials.gov).

Cdk inhibitors (Alias(es))	Generation	Principal antikinase activity	Clinical trials for the treatment of tumors
Seliciclib (CYC-202;R-roscovitine)	I	Cdk1, 2, 5, 7, 9; CK1; GSK3A; DIRK1A; ERK1	Phases I-II for nonsmall cell lung cancer (NSCLC) and other solid tumors

Alvocidib (flavopiridol)	I	Cdk1, 2, 4, 6, 7, 9; GSK3*β*	Phases I-II for various types of cancers, such as multiple myeloma, leukemia, lymphomas, sarcoma, and solid tumors

Dinaciclib (SCH727965)	I	Cdk1, 2, 5, 9	Phases I-II for various solid tumors; phases I-II for acute myelogenous leukemia, acute lymphoblastic leukemia, mantle cell lymphoma, and B cell chronic lymphocytic leukemia

SNS-032 (BMS-387032)	I	Cdk1, 2, 4, 7, 9	Phases I-II for B-cell malignancies, nonsmall cell lung cancer (NSCLC), advanced breast cancer, and melanoma

AG-024322	I	Cdk1, 2, 4, 7	Phase I for non-Hodgkin's lymphoma and advanced solid tumors

R547 (R_0_-4584820)	I	Cdk1, 2, 4, 7	Phase I for advanced solid tumors

P276-00	II	Cdk1; Cdk4; Cdk9	Phases I-II for multiple myeloma and various advanced refractory malignancies

PD-0332991	II	Cdk4, Cdk6	Phase I for non-Hodgkin's lymphoma, mantle cell lymphoma, and other malignancies

AT-7519	II	Cdk2, Cdk4, Cdk5, Cdk9; GSK3*β*	Phases I-IIa for advanced and/or metastatic solid tumors and refractory non-Hodgkin's lymphoma

RGB-286638	II	Cdk1, Cdk2, Cdk4, Cdk5, Cdk7, Cdk9	Entering a phase I clinical trial for the treatment of advanced solid tumors

ZK 304709	III	Cdk1, 2, 4, 7, 9; VEGFR1, 2, 3; PDGFR-b; Flt-3	Phase I trials for refractory and/or relapsed solid tumors

GPC-286199 (RGB-286199**)**	III	Cdk1, 2, 3, 5, 7, 9; CRKs	Preclinical stage

JNJ-7706621	III	Cdk1, 2, 3; Aurora A/B	Preclinical stage
